# Adenoviral Delivery of the EMX2 Gene Suppresses Growth in Human Gastric Cancer

**DOI:** 10.1371/journal.pone.0045970

**Published:** 2012-09-21

**Authors:** Jie Li, Minli Mo, Zhao Chen, Zhe Chen, Qing Sheng, Hang Mu, Fang Zhang, Yi Zhang, Xiu-Yi Zhi, Hui Li, Biao He, Hai-Meng Zhou

**Affiliations:** 1 Protein Science Laboratory of Ministry of Education, School of Life Sciences, Tsinghua University, Beijing, China; 2 College of Life Sciences, Zhejiang Sci-Tech University, Hangzhou, China; 3 Zhejiang Provincial Key Laboratory of Applied Enzymology, Yangtze Delta Region Institute of Tsinghua University, Jiaxing, Zhejiang, China; 4 Xuanwu Hospital, Capital Medical University, Beijing, China; 5 Department of Surgery, Helen Diller Family Comprehensive Cancer Center, University of California San Francisco, San Francisco, California, United States of America; National Cancer Center, Japan

## Abstract

**Background:**

EMX2 is a human orthologue of the *Drosophila* empty spiracles homeobox gene that has been implicated in embryogenesis. Recent studies suggest possible involvement of EMX2 in human cancers; however, the role of EMX2 in carcinogenesis needs further exploration.

**Results:**

In this study, we reported that down-regulation of EMX2 expression was significantly correlated with EMX2 promoter hypermethylation in gastric cancer. Restoring EMX2 expression using an adenovirus delivery system in gastric cancer cell lines lacking endogenous EMX2 expression led to inhibition of cell proliferation and Wnt signaling pathway both in vitro and in a gastric cancer xenograft model in vivo. In addition, we observed that animals treated with the adenoviral EMX2 expression vector had significantly better survival than those treated with empty adenoviral vector.

**Conclusion:**

Our study suggests that EMX2 is a putative tumor suppressor in human gastric cancer. The adenoviral-EMX2 may have potential as a novel gene therapy for the treatment of patients with gastric cancer.

## Introduction

Gastric cancer is the fourth most common cancer and second most common cause of cancer-related deaths in the world [1], [2], [3]. Although its global incidence has declined, the incidence still remains high in Asian countries [2], [4], [5]. Most of gastric cancer patients are diagnosed at advanced stages [6]. Despite of improvement in treatment strategies [7], [8], 5-year survival of patients with advanced gastric cancer who received conventional therapies remains less than 30% [4]. Therefore, alternative approaches are urgently needed.

Gene therapy, including viral and non-viral gene delivery system, is a promising approach for cancer treatment [9]. Among all currently available system, recombinant adenovirus (Ad) vectors are particularly promising. The advantages of Ad vectors include the ability to produce high titers, ability to infect dividing and non-dividing cells efficiently, genome stability, and low levels of DNA integration into host genome [9], [10]. It has been reported that restoration of genes through this approach leads to tumor regression and induces apoptosis *in vivo* without affecting normal tissues [11], [12], [13].

EMX2, the human ortholog of the *Drosophila* empty spiracles (ems) homeobox gene, belongs to the Homeobox gene family which encodes transcriptional regulatory proteins (Homeoprotein) essential for many aspects of growth and differentiation [14]. EMX2 plays a vital role during brain [15] and skeletal development [16]. Mice harboring homozygous EMX2 mutations exhibit dramatic size reduction of caudal-medial areas including occipital cortex and hippocampus [17], [18], [19]. Perturbation of EMX2 expression can alter the proliferation rate of adult neural stem cells [20]. EMX2 also controls mammalian reproduction by adjusting endometrial cell proliferation without effecting differentiation [21].

Recent studies suggest possible involvement of EMX2 in human cancers [22], [23], [24], [25]. For example, EMX2 has been demonstrated as a tumor suppressor in lung cancer, and low EMX2 expression is associated with decreased overall survival and recurrence free survival in patients with lung adenocarcinomas [22], [23]. It is also reported that EMX2 expression is abundant in normal endometrium of postmenopausal women but absent or reduced in endometrial tumors, and EMX2 levels are negatively correlated with proliferation [24], [25]. Moreover, EMX2 may regulate tumorigenesis through Wnt signaling pathway, a critical pathway regulating cell fate determination, tissue development and tumorigenesis [26], [27], [28]. EMX2 is found as a direct repressor of Wnt1 expression, and knocking-down of EMX2 gene induces ectopic expression of Wnt1 in the developing telencephalon and cortical dysplasia [29]. Epigenetic silencing of the EMX2 expression may be important for aberrant activation of the Wnt signaling in lung cancer, and consequent proliferation and metastasis [22]. However, the function of EMX2 during tumorigenesis and corresponding mechanism still need to be further elucidated. In this study, we report EMX2 as a putative tumor suppressor in human gastric cancer. We demonstrate the EMX2 downregulation and its correlation with methylation status of the gene promoter region in gastric cancer. We also show that restoration of EMX2 using an adenoviral delivery system inhibits cell proliferation and Wnt signaling *in vitro*, and results in better survival of a gastric cancer xenograft model *in vivo*. Our data strongly suggest the therapeutic potential of using the Ad-EMX2 as gene therapy for future treatment of patients with gastric cancer.

## Materials and Methods

### Ethics Statement

This study was approved by the ethics committee of Xuanwu Hospital, Capital Medical University in Beijing. A written informed consent approved by the ethics committee, was obtained for each patient prior to tissue specimen collection.

### Cell Culture, 5-aza-2'-deoxycytidine (DAC)/trichostatin A (TSA) Treatment, and Tissue Samples

Human gastric cancer cell lines (AZ521, AGS and MKN28) were obtained from China Center for Type Culture Collection (Wuhan, China). Cell lines were cultured in Dulbecco’s modified Eagle’s medium supplemented with 10% fetal bovine serum, penicillin, and streptomycin at 37°C in a humid incubator with 5% CO_2_. To analyze restoration of EMX2 gene, cells were treated with 4 µM DAC or 300 nM TSA (Sigma, St. Louis, USA) for 4 days, with replacement of fresh medium containing the same dose of DAC or TSA everyday. Control cells were cultured with fresh medium containing DMSO. Cells were consequently harvested for DNA/RNA extraction.

A total of 20 formalin-fixed paraffin-embedded tissues blocks (10 gastric cancer tissues and 10 adjacent normal tissues), as well as total RNA from 15 gastric dysplasia samples and 20 gastric cancer surgical samples were obtained from Xuanwu Hospital, Capital Medical University in Beijing. Total RNA and genomic DNA extracted from human adult normal gastric tissues were purchased from Biochain (Hayward, CA, USA).

### DNA Constructs

Topflash/Fopflash reporters containing wild type and mutant TCF/LEF binding sites respectively were kindly provided by Dr. Yeguang Chen (Tsinghua University, Beijing, China). A mutant CTNNB1 (S45Y) cDNA construct was also kindly provided by Dr. Yeguang Chen (Tsinghua University, Beijing, China). Recombinant adenovirus vectors expressing EMX2 and the control vector were purchased from Vector Biolabs (Vector Biolabs, Burlingame, CA, USA).

### Methylation-specific PCR (MSP) and Bisulfite Sequencing (BS)

The bisulfite conversion from tissues and cells were performed without the prerequisite for DNA purification using EZ DNA Methylation-Direct Kit (Zymo, Orange, CA, USA). The FFPE tissues were first de-paraffinized in xylene (Sigma) and rehydrated in graded ethanol, before bisulfite treatment per manufacture’s instructions. For MSP, modified DNA is amplified using MSP primers (listed below) which specially recognized either the methylated or unmethylated EMX2 promoter sequences after bisulfite treatment. PCR was run in a final volume of 20 µl containing 1×MSP reaction buffer [30], 0.5 µM of each primer and 1 U of Zymo*Taq*™ DNA Polymerase (Zymo). PCR condition was as follows: 10 min at 95°C; 40 cycles of 30 s at 95°C, 30 s at 50°C and 30 at 72°C; and 7 min final extension at 72°C. For BS, amplification of bisulfite-converted DNA was performed in a final volume of 50 µl containing 1 µM each BS primer and 2 U of Zymo*Taq*™ DNA Polymerase. PCR condition was as follows: 10 min at 95°C; 40 cycles of 30 s at 95°C, 40 s at 55°C and 1 min at 72°C; and 7 min final extension at 72°C. The PCR products were cloned into pMD-18T (Takara, Dalian, China). Either 5 or 3 randomly selected positive clones from each group were sequenced in Shanghai Invitrogen (Shanghai, China). Primers were as follows:

MSP-M (forward): 5′- TAGTTTTTTGTTCGTTTCGCGTTTC-3′

MSP-M (reverse): 5′- GAATTAAAATAAACGCCCCTACCGAC-3′

MSP-U (forward): 5′-GTTTTTTGTTTGTTTTGTGTTTTGA-3′

MSP-U (reverse): 5′-CCAAATTAAAATAAACACCCCTACCAAC-3′

BS-A (forward): 5′-GTTTGTAAATTTTTTTGGAAGGATTT-3′

BS-A (reverse): 5′-AACAAAAAACTATCCTTAACCACCA-3′

BS-B (forward): 5′-GGTTAGGGATTTTGTAGGGAT-3′

BS-B (reverse): 5′-AAAATCACATAAACAACTTCCTCC-3′

### Immunohistochemistry (IHC)

IHC was performed following standard protocol. Antibodies used in the study included mouse anti-human Ki67 (1∶100; BD, San Jose, CA, USA) and mouse anti-human EMX2 (1∶500; Pierce, Rockford, IL, USA), and Alexa 594-conjugated goat anti-mouse secondary antibody (1∶200; Invitrogen, Carlsbad, CA, USA). Slides were also counterstained with hochest 33342 (Invitrogen). Zeiss LSM 710 confocal microscope (Oberkochen, Germany) was used to examine the staining. The intensity of Ki67 was quantified using Image Pro® Plus. EMX2 staining was visualized with Histostain Plus DAB kit (broad spectrum, Invitrogen) according to manufacture’s instructions, counterstained with hematoxylin (Sigma), and photographed using a Leica SCN400 slide scanner (Leica, Germany).

### Luciferase Assay

Cells at a density of 5×10^3^ per well were seeded into 24-well plates before transfection. Topflash or Fopflash plasmids were co-transfected with PRL-TK plasmid. After cells were cultured for 18 hours, the luciferase activity was measured by Dual-Glo Luciferase Assay System (Promega, Madison, USA). The ratio between firefly luciferase activity (Topflash/Fopflash) and renilla activity was used for TCF/LEF transcription activity.

### Immunoblotting

Both total protein (20 µg) and cytoplasm extracts (40 µg) were subjected to immunoblotting. The primary antibodies included anti-EMX2 (1∶500; Pierce), anti-β-Actin (1∶5000; Sigma), anti-Cyclin D1 (1∶500; BD) and anti-c-Myc (1∶200; Santa Cruz Biotechnology, CA, USA).

### Proliferation Assay

Cell growth was determined by CellTiter 96 Aqueous Proliferation Assay Kit (Promega). The cell lines were plated into 96-well culture plates with the number of 5×10^2^ cells/well. After the cells were cultured for days 0, 2, 3 and 4, the MTS solutions were added to the medium and incubated for 1.5 h. The absorbance at 490 nm was measured using microplate reader model 680 (Bio-Rad, Hercules, CA, USA).

### Colony Formation Assay

Cells (1×10^3^) infected with adenoviral vector expressing EMX2 or empty vector were seeded in triplicate into 100-mm dishes. Fresh medium was changed every 3 days. After cultured for 3–4 weeks, cells were fixed with 4% paraformaldehyde for 10 min, washed with PBS, stained with 0.1% crystal violet for 20 min and photographed.

### RNA Extraction and Quantitative Real-time Reverse Transcription-PCR (RT-PCR)

RNA extraction and real-time RT-PCR were performed as previously described [31]. The primer sequences were previously described [22].

### Animal Model and Adenoviral Vector Delivery

All mice experiments were conducted under specific pathogen-free conditions in animal facility of Tsinghua University and approved by the Institutional Animal Care and Use Committee of Tsinghua University. Gastric cancer xenografts were established into 5-week-old female BALB/c nude mice. Briefly, after cultured to confluence, MKN28 or AGS cells were trypisnized and resuspended in PBS (pH 7.4) and then mixed 1∶1 (v/v) with matrigel (Vigorous) at 4°C to inject one mouse in a total volume of 150 µl. The mixture was s.c injected into right flank of 16 female mice with 10^7^ cells per mouse (day 0). On day 7, mice bearing local tumors ranged from 50 to 100 mm^2^ received a direct intra-tumoral injection of 1×10^9^ plaque-forming units of the indicated adenovirus (diluted with PBS in a total volume of 100 µl). The tumor formation was monitored for up to 1½ months. Tumor size was measured using caliper and determined by multiplying by 0.5×width^2^×length. The Kaplan-Meier method was used to estimate survival of the two groups of treated animals. At completion of the experiment, the tumors from each treatment were collected in 10% buffered formalin, embedded in paraffin and cut into 5-µm thick slices for further Ki-67 detection. Cytosolic proteins and whole cell lysate were also extracted from those tumors for Western analysis.

### Statistical Analysis

All data were calculated as means ± standard deviations. Differences between groups were compared with a two-sided student’s t-test. A *P* value of 0.05 or less was considered to be significant.

## Results

### Downregulation of EMX2 in Human Gastric Cancer

**Figure 1:**
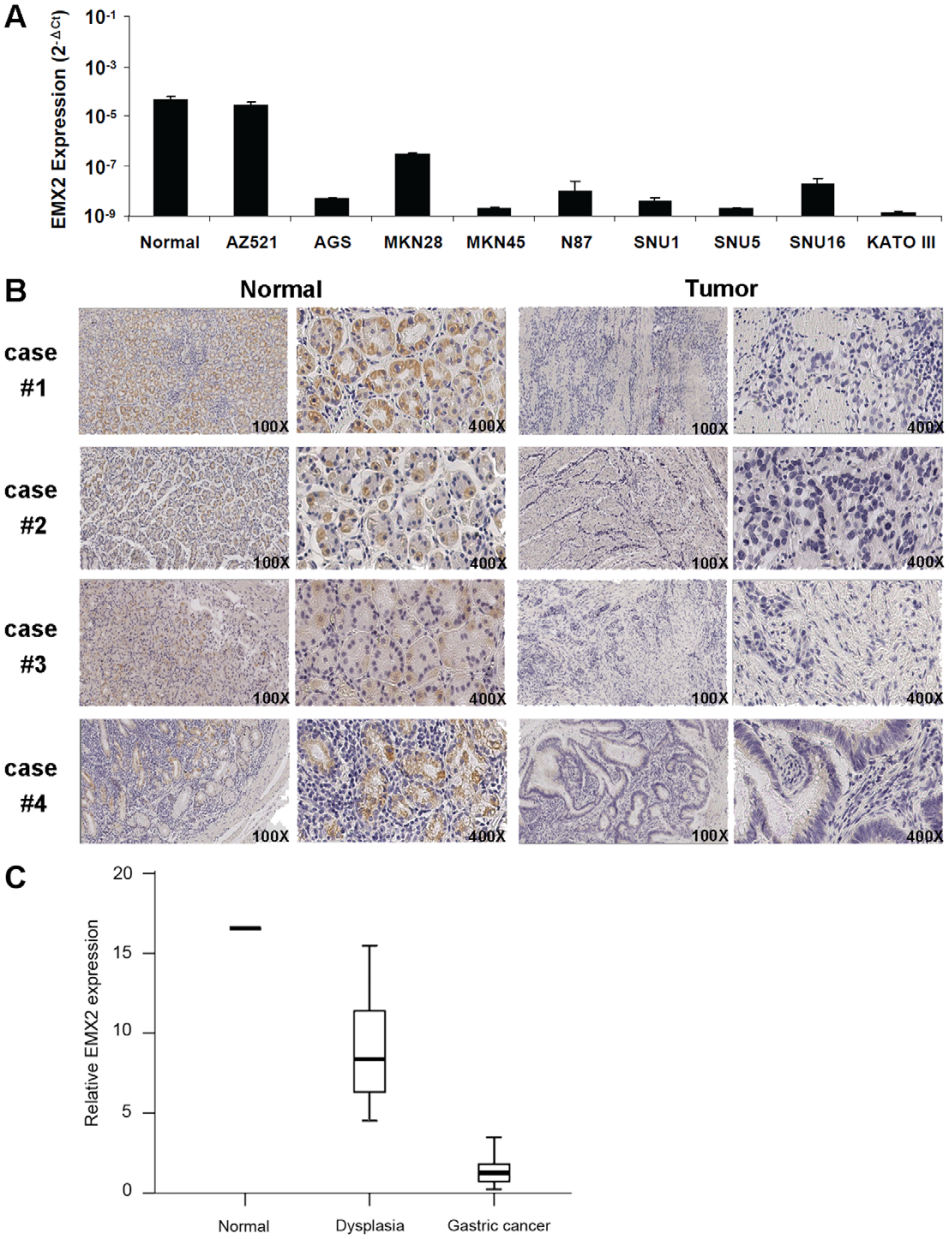
Downregulation of EMX2 expression in human gastric cancer. (A) Real-time RT-PCR of EMX2 expression in a normal stomach tissue sample and gastric cancer cell lines. (B) IHC staining of EMX2 protein in gastric cancer tissues and their adjacent normal tissues from the same patients. (100X, scale bar = 500 µm; 400X, scale bar = 100 µm). (C) Real-time RT-PCR result of 15 gastric dysplasia and 20 gastric cancer surgical samples. An adult normal stomach tissue sample was used as a control. 25th and 75th percentiles are represented as box margins, 10th and 90th percentiles are represented as error bars, and the median is represented as a line in the box.

We examined EMX2 expression in nine human gastric cancer cell lines, as well as tumor and paired adjacent normal tissues from ten gastric cancer patients (Figure 1). Using real time RT-PCR, we found that expression of EMX2 was significantly downregulated in eight of the nine cell lines examined when compared to that in normal gastric tissue (P<0.01, Figure 1A). On the other hand, one cell line AZ521 showed similar level of EMX2 expression as that in normal control (Figure 1A). In order to detect EMX2 protein expression in patient tissue samples, immunohistochemistry (IHC) was performed and the intensity of IHC staining was quantified by Image Pro® Plus. All ten samples analyzed were found to exhibit either lack of EMX2 expression or decreased levels of EMX2 expression in cancer tissues when compared to their normal counterparts (P<0.01, Figure 1B). To establish the possible role of EMX2 in pathological progression of human gastric cancer, we analyzed EMX2 expression using total RNA that we obtained from fifteen gastric dysplasia samples and twenty gastric cancer surgical samples. We found that EMX2 expression was significantly downregulated in dysplasia samples (P<0.05) and almost lost in cancer samples compared to that in adult normal stomach tissue (Figure 1C), suggesting that EMX2 downregulation could occur at non-invasive precancerous stage.

### Correction between EMX2 Expression and its Promoter Methylation Status

**Figure 2:**
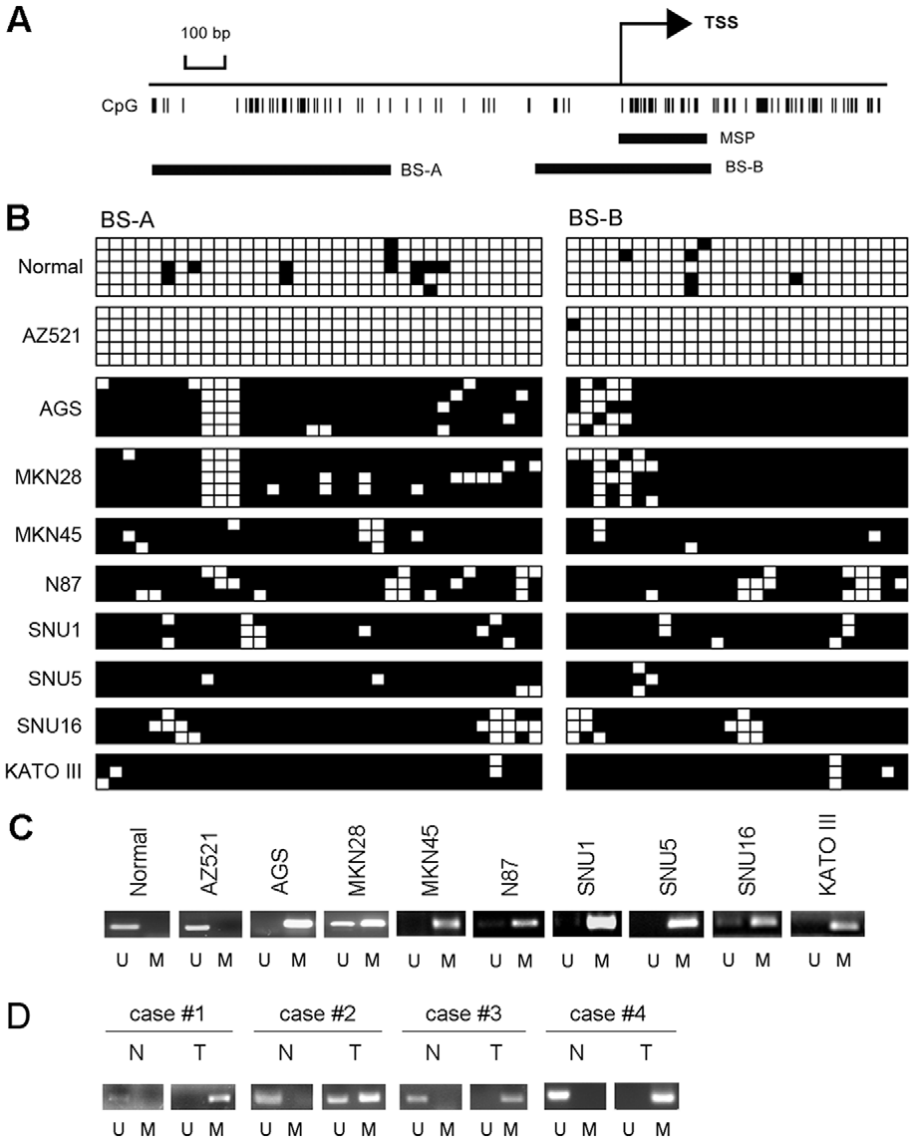
Correlation of EMX2 downregulation and promoter hyper-methylation in human gastric cancer. (A) Schematic representation of the 5′ promoter region of EMX2 gene. TSS indicates the transcriptional start site. MSP is the MSP amplicon, and BS-A/BS-B are amplicons for bisulfite sequencing. (B) Bisulfite sequencing results of the normal stomach tissue sample and gastric cancer cell lines. Open and filled squares represent the unmethylated and methylated sites respectively. (C) MSP results of the EMX2 promoter region in normal stomach tissue sample and gastric cancer cell lines. (D) MSP results of the EMX2 promoter region in gastric cancer tissues and their matched adjacent normal tissues.

We examined methylation status of the EMX2 promoter in nine gastric cancer cell lines and normal gastric tissue. Regions of CpG islands (CGI) were identified with MethPrimer from −800 to −470 and −55 to 459 relative to the expected transcription start sites (+1) of EMX2 gene (Figure 2A). Methylation status was analyzed by using bisulfite sequencing (BS) (Figure 2B) and methylation-specific PCR (MSP) (Figures 2C–2D). Our BS and MSP results showed that the EMX2 promoter in normal gastric tissue and AZ521 cells was unmethylated, whereas in the other eight cell lines examined it was densely methylated (Figures 2B–2C). These results are consistent with the EMX2 expression levels in these cell lines: high in normal tissue and AZ521, but low in the other eight cell lines examined. A mixture of methylated and unmethylated band was found in several cell lines including MKN28, N87, and SNU16 indicating partial methylation. Furthermore, we found that methylation status of the patient tissue samples revealed by MSP is consistent with the EMX2 expression levels in those samples (Figure 2D, four of the ten pairs of tissue samples were examined due to unavailability of genomic DNA from the other six pairs). These data suggest that downregulation of EMX2 expression may be a result of its promoter hyper-methylation in gastric cancer.

**Figure 3:**
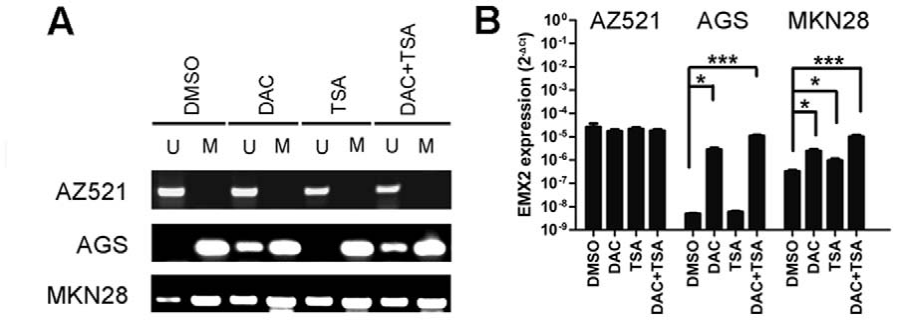
Correlation of EMX2 downregulation and promoter hyper-methylation in human gastric cancer. MSP (A) and Real-time RT-PCR (B) analyses in gastric cancer cell lines after treatment of DMSO, 1 µM DAC, 300 nM TSA, and a combination of DAC (1 µM) and TSA (300 nM) respectively (*: *P<0.05*, ***: *P<0.001*).

To further investigate the correlation between EMX2 expression and the promoter methylation, we treated MKN28 and AGS cell lines with a methyltransferase inhibitor DAC and a histone deacetylase inhibitor TSA. Cell line AZ521 was used as a negative control as its promoter region is unmethylated. We observed that the unmethylation specific band was significantly increased (Figure 3A) with EMX2 expression levels upregulated accordingly (Figure 3B) upon DAC treatment in both AGS and MKN28 cells, whereas those in AZ521 cells remained unchanged (Figure 3). The treatment of TSA had minimal effects on both methylation status and EMX2 expression levels. Taken together, our results strongly suggest the down-regulation of EMX2 expression was significantly correlated with the EMX2 promoter hypermethylation in human gastric cancer.

### Inhibition of Cell Growth by Adenovirus-delivered EMX2 in vitro

**Figure 4:**
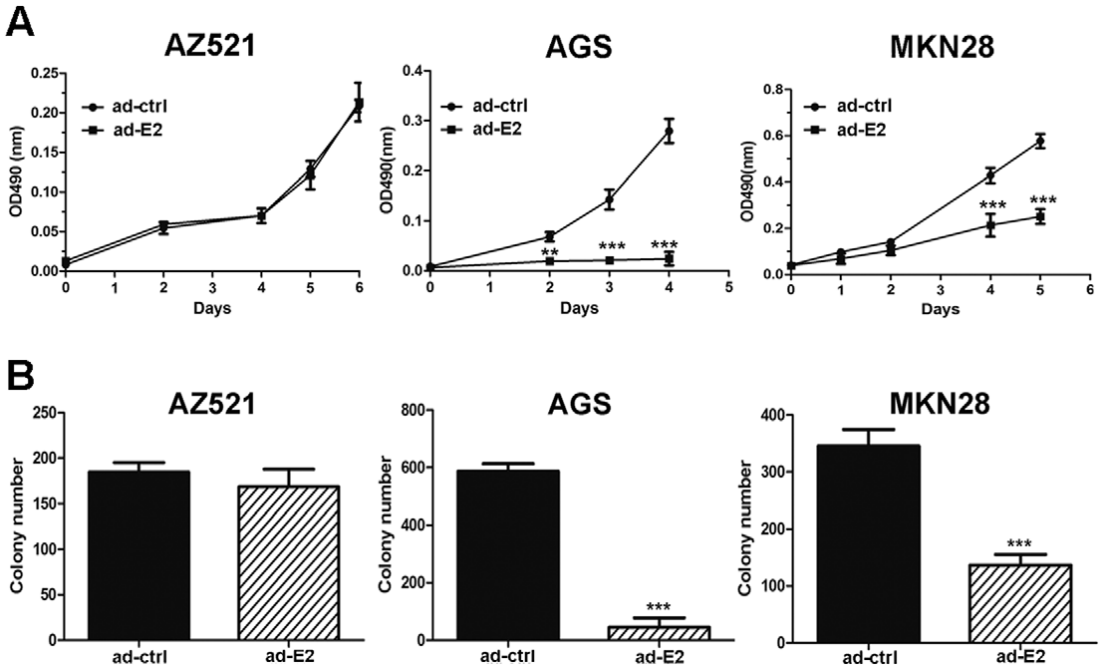
Restoration of EMX2 expression using an adenoviral delivery system suppressed gastric cancer cell proliferation *in vitro*. (A) MTS assay. (B) Quantification of colony formation assay (**: *P<0.01*, ***: *P<0.001*). ad-ctrl and ad-E2 represent the empty adenoviral vector control and the adenoviral vector containing EMX2 cDNA, respectively.

We next examined the role of EMX2 on cell growth of gastric cancer cells. The growth of AGS and MKN28 cells was significantly suppressed upon infection with adenovirus expressing EMX2 (Ad-EMX2) compared with an empty vector control (Ad-ctrl) (P<0.001, Figure 4A), whereas the growth of AZ521 cells was not affected (P>0.05, Figure 4A). These observations were also confirmed by colony formation assay (Figure 4B). Our results demonstrate the anti-proliferation function of EMX2 in gastric cancer cells.

**Figure 5:**
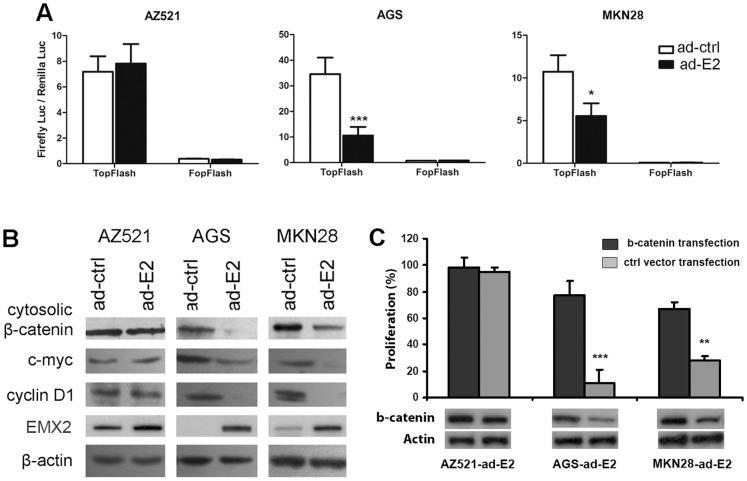
Restoration of EMX2 expression using an adenoviral delivery system suppressed canonical Wnt signaling in gastric cancer cell lines. TCF/LEF reporter activity analysis (A), and Western blot of EMX2, cytosolic β-catenin and canonical Wnt pathway target genes (B) in gastric cancer cell lines infected with ad-ctrl or ad-E2. (C) Effect of proliferation suppression induced by ad-EMX2 after a stabilized β-catenin transfection. 0.5 µg mutant CTNNB1 (S45Y) cDNA or an empty vector control was used in each transfection. Cell proliferation was assessed at day 5 after transfection and infection. Western was to confirm expression of the mutant β-catenin (*: *P<0.05*, **: *P<0.01,* ***: *P<0.001*).

### Suppression of Wnt Signaling Pathway by Adenovirus-delivered EMX2

The function of EMX2 has been linked to Wnt signaling pathway; therefore we studied the relationship between EMX2 and Wnt signaling in gastric cancer. We used a Topflash/Fopflash reporter assay to measure TCF/LEF-dependent transcription activity regulated by canonical Wnt pathway. We found that TCF/LEF-dependent transcription activity was significantly inhibited by Ad-EMX2 in AGS and MKN28 cells lacking endogenous EMX2 expression (P<0.05), but not in AZ521 cells expressing endogenous EMX2 (P>0.05, Figure 5A). Consistently, protein levels of cytosolic β-catenin and canonical Wnt pathway downstream targets c-myc and cyclin D1 were suppressed in these AGS and MKN28 cells infected with Ad-EMX2, but not in AZ521 cells (restoration of EMX2 expression in these cell lines after Ad-EMX2 infection was confirmed by Western) (Figure 5B). To further examine the relevance of Wnt pathway down-regulation and proliferation suppression by Ad-EMX2, we transfected and expressed stabilized β-catenin (S45Y mutation) in these gastric cancer cells infected with Ad-EMX2. We observed that over-expressing β-catenin significantly attenuated the growth suppression effect of Ad-EMX2 in both AGS and MKN28 cells (P<0.01), but not in AZ521 cells (Figure 5C). Taken together, these results support an important role of EMX2 in suppression of Wnt pathway in gastric cancer.

**Figure 6:**
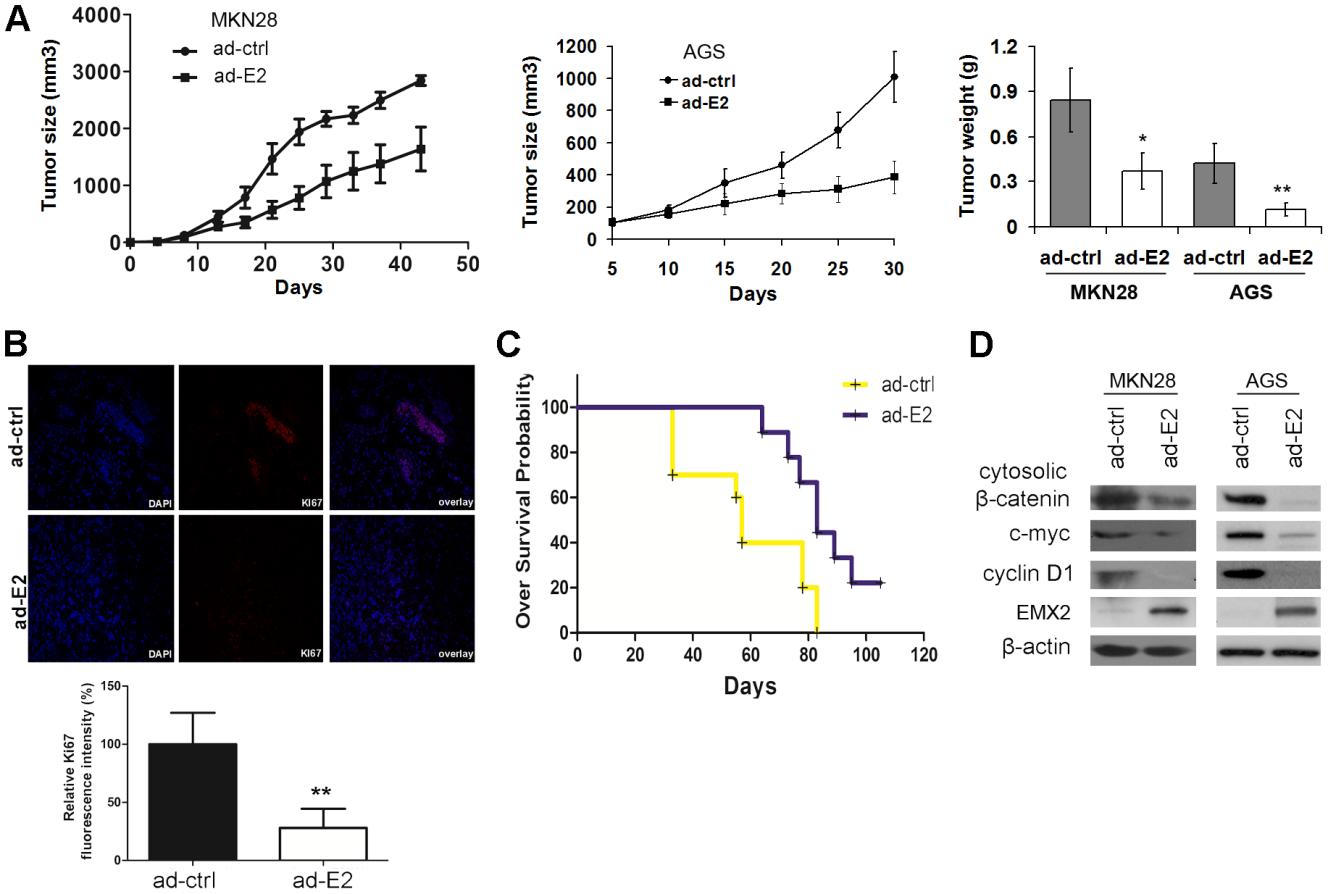
Adenoviral delivery of EMX2 suppressed gastric tumor growth in vivo. (A) Tumor volume and tumor weight of gastric cancer xenograft models (MKN28 and AGS) treated with ad-ctrl or ad-E2. (B) Images of Ki-67 staining of MKN28 tumors treated with ad-ctrl or ad-E2 (upper panel) and quantification of the staining (lower panel). (C) Kaplan-Meier estimates for cumulative survival of mice treated with ad-ctrl or ad-E2 (**: *P<0.01*). (D) Western blot of EMX2, cytosolic β-catenin and canonical Wnt pathway target genes in MKN28 and AGS tumors treated with ad-ctrl or ad-E2.

### Suppression of Gastric Cancer Growth by Adenovirus-delivered EMX2 in vivo

We established the MKN28 and AGS xenograft models to explore the therapeutic potential of adenovirus-delivered EMX2 for gastric cancer *in vivo*. One week after inoculation, mice bearing local tumors ranging from 50 to 100 mm^2^ received a direct intratumoral injection of 1×10^9^ plaque-forming units of the indicated adenovirus. Growth of tumors in Ad-EMX2 infected mice were significantly slower than that in control group (P<0.05, Figure 6A left two panels). At completion of the experiment tumor mass in Ad-EMX2 infected mice also significantly less than that in control group (P<0.05 for MKN28 tumor, P<0.01 for AGS tumor, Figure 6A right panel). Staining intensity of a proliferative marker Ki67 of tumor specimens at completion of the experiment showed that delivery of Ad-EMX2 significantly diminished the Ki-67 staining (P<0.01, Figure 6B), suggesting cell proliferation inhibition in tumors. Moreover, survival of the Ad-EMX2 infected group was significantly better than that of the control group (P = 0.014, Figure 6C). Consistent with our *in vitro* analysis, cytosolic β-catenin and canonical Wnt pathway downstream targets c-myc and cyclin D1 were also downregulated in Ad-EMX2 infected MKN28 and AGS tumors (restoration of EMX2 expression in these in vivo tumors was also confirmed by Western) (Figure 6D). Taken together, our results demonstrate a therapeutic potential of adenovirus-delivered EMX2 to treat gastric cancer.

## Discussion

Homeobox genes have been well known for its importance in development for decades, while the study of these genes during oncogenesis is still in its infancy. Aberrant homeobox gene expressions have been documented in various cancers [32], [33], indicating the changes of hemeobox expressions might be important for oncogenesis. This may provide a molecular basis for potential clinical applications. However, several fundamental questions still need to be fully addressed, including the molecular mechanisms that drive the aberrant expression, and downstream targets and signaling pathways that promote oncogenesis. In this study, we provide insights on these questions by investigating EMX2 in human gastric cancer.

Our study reported a significant decrease and loss of EMX2 expression in gastric cancer cell lines and primary tumor tissues, and showed that the downregulation was significantly correlated with hyper-methylation of the EMX2 promoter, suggesting epigenetic silencing as an important mechanism for EMX2 dysregulation in human gastric cancer. Indeed, epigenetic modification has been proposed as a key mechanism responsible for homeobox genes downregulation or silencing in other cancer tissue types where these genes function in tumor suppression [14], [32]. Moreover, our observation of EMX2 dowregulation in non-invasive gastric dysplasia supports a possible important role of EMX2 in pathological progression of human gastric cancer. One limitation of our study is the number of tissue samples analyzed. A larger number of patient samples need to be examined to further validate the finding of methylation-silencing of EMX2 in gastric cancer. Nevertheless, our results provide a first direct evidence to support this mechanism in gastric cancer and identify EMX2 as a putative novel tumor suppressor in gastric cancer.

In addition, we investigated important oncogenic pathways through which EMX2 functioned in gastric cancer, and found that Wnt signaling pathway may play a key role mediating the EMX2 function. We illustrated in proliferation assays that high expression of exogenous EMX2 significantly suppressed growth of gastric cancer cell lines lacking endogenous gene expression (AGS and MKN28 cells), consistent with previous reports that difference in EMX2 expression is negatively correlated with proliferation in other cancer cell types [24], [25]. We also demonstrated that Wnt signaling pathway was dramatically inhibited by EMX2 *in vivo* and *in vitro*, providing further evidence that Wnt signaling pathway may mediate the function of EMX2 in cancer as previously proposed [22].

Finally, our results indicate the potential of using EMX2 gene therapy for the treatment of gastric cancer. Infection of tumors with Ad-EMX2 significantly suppressed proliferation and more importantly, improved overall survival. It is noteworthy that the delivery system we used for the treatment was recombinant adenovirus serotype 5 (Ad5), the most frequently used vector in several types of cancer gene therapy [11], [12], [34], [35]. Compared with adeno-associated viral (AAV) and retroviral delivery systems, adenoviral vectors, such as Ad5, clearly have many advantages. For example, adenoviral vectors have broad tropism allowing efficient targeting on many tissues of interest, a large payload capacity and high transduction efficiency relative to AAV system [10] and also a non-integrating characteristics which otherwise induce the risk of random mutagenesis of genome [10], [36] [36], [37], [38]. Moreover, adenoviral vectors can be engineered to cancer-selective oncolytic viruses [39], [40], [41], which are critical for clinical application of cancer gene therapy. However, there are still many challenges of using adenoviral vectors to deliver gene therapies in patients. For example, they can be quickly lost from cells that divide rapidly after infection. Other factors affecting clinical application of adenoviral delivery include packaging capacity and host range of adenoviral vectors, their gene expression profile and tendency to elicit immune responses, particularly important if repeated administration is needed [42], [43]. Nevertheless, our *in vivo* study of Ad-EMX2 infection suggests that EMX2 gene therapy may have potential to become a clinical anti-tumor therapeutic strategy for the treatment of gastric cancer in the future.
